# Design, Modeling, and Experimental Verification of a Fully Decoupled Tendon-Driven Humanoid Arm

**DOI:** 10.3390/biomimetics11020141

**Published:** 2026-02-12

**Authors:** Diwei Huang, Hao Li, Xiao Jiang, Jiahao Shen, Hong Luo, Chongkun Xia, Xueqian Wang

**Affiliations:** 1 School of Advanced Manufacturing, Sun Yat-sen University, No. 66, Gongchang Road, Guangming District, Shenzhen 518107, China; huangdw8@mail2.sysu.edu.cn (D.H.); lihao279@mail2.sysu.edu.cn (H.L.); jiangx223@mail2.sysu.edu.cn (X.J.); shenjh6@mail2.sysu.edu.cn (J.S.); 2 Shenzhen International Graduate School, Tsinghua University, Shenzhen 518055, China; luo-h23@mails.tsinghua.edu.cn (H.L.); wang.xq@sz.tsinghua.edu.cn (X.W.)

**Keywords:** tendon-driven humanoid arm, joint space decoupling, antagonistic

## Abstract

Human upper-limb movement is produced through the antagonistic action of tendons and is controlled in a joint space-oriented manner. Inspired by this functionality, a fully decoupled tendon-driven humanoid arm (FDTDH-Arm) is proposed, in which joint space decoupling is achieved at the mechanical level via humanoid antagonistic actuation and joint regulation rather than complex modeling-based compensation. To characterize the motion behavior introduced by rolling constraints, joint-level and whole-arm kinematic models are established. A prototype of the proposed arm is developed and experimentally validated. The results demonstrate effective mechanical joint space decoupling, passive joint stiffness of the same order of magnitude as that reported for the human upper limb, a mean positioning error of 0.40 mm, and rapid whole-arm motion with a maximum end effector velocity of 3.62 m/s. The proposed design provides a mechanical implementation and biomimetic solution for humanoid manipulation in human-interactive environments.

## 1. Introduction

Robotic arms are increasingly evolving from traditional unmanned industrial settings toward unstructured, human-interactive environments. This transition has stimulated extensive research on humanoid robotic arms that emphasizes lightweight construction and high dexterity [1,2,3]. However, most existing humanoid arms remain primarily biomimetic in appearance, while their mass, scale, and safety characteristics are still closer to those of conventional industrial manipulators [4,5]. Consequently, a substantial gap persists between these systems and the human arm in terms of their functional and mechanical properties, limiting their applicability in humanoid-interactive applications. In the human upper-limb system, joint motion is generated through the coordinated contraction and extension of tendons. Tendon-driven actuation has been shown to effectively emulate this muscle–tendon paradigm [6,7]. Compared with traditional gear-driven actuators, tendon-driven mechanisms inherently offer enhanced compliance [8,9], thereby improving safety [10,11] and adaptability during physical interaction [12,13]. Owing to these advantages, tendon-driven mechanisms have demonstrated strong potential in lightweight safe services [14], human–robot interaction [15], unmanned nuclear power plant maintenance [16], and interactive spacecraft operations [17].

A growing body of research has therefore focused on tendon-driven humanoid arms (TDH-Arms). In particular, a clear research trend has emerged toward reducing on-link actuation to decrease arm mass and redistribute inertia, with the aim of achieving human-like rotational inertia and dynamic behavior. Kim et al. introduced a TDH-Arm for high-speed and safe human–robot interaction [18], integrating a tension-amplifying decoupling joint to achieve stiffness comparable to that of conventional industrial robotic arms [19,20]. However, the four motors actuating the elbow and wrist are still integrated at the base of the forearm, resulting in a relatively high link mass of 5.84 kg, which is larger than a human arm (approximately 3 kg) [21]. Huang et al. further designed a hybrid serial–parallel manipulator featuring a high-stiffness parallel shoulder joint [22]. The use of rigid link-based parallel joints, however, leads to relatively low overall compliance of the arm. The primary reason why the aforementioned TDH-Arms have difficulty in further reducing link mass, and consequently achieving rotational inertia and motion performance closer to those of the human arm, lies in the inherent motion-coupling effects of TDH-Arms [23]. Owing to the high density of degrees of freedom at the shoulder, it is difficult to maintain invariant tendon lengths for the elbow and wrist during shoulder motion.

To overcome the above limitations, researchers have proposed several solutions. For example, Wang et al. proposed a modular tendon-driven humanoid arm that achieves complete motor–link separation by directly routing Bowden tendons to the joints [24], reducing the mass of the moving components to only 2.17 kg. Nevertheless, Bowden tendon friction limits the positioning repeatability to 1.5 mm. Suzuki et al. developed a low-friction passive 3D wire aligner that guides tendons directly to actuated joints, thereby enabling complete motor–link separation [25]. Similarly, Tanaka et al. implemented a pulley-based routing strategy in a serial TDH-Arm [26]. However, both approaches rely on algorithmic compensation to achieve kinematic decoupling, which constrains positioning accuracy due to the nonlinear effects inherent in tendon transmission. Luo et al. proposed a 6-DOF tendon-driven robotic arm achieving motor–link separation [27]. However, the wrist-driving tendons pass directly through guide holes, introducing significant friction, which adversely affects positioning accuracy. Moreover, the wrist adopts a 2-DOF parallel platform configuration, resulting in a relatively complex kinematic mapping. In summary, existing tendon-driven humanoid arms continue to face a trade-off between achieving kinematic decoupling and motor–link separation, and therefore fall short of faithfully reproducing the dynamic and inertial characteristics of the human arm.

Inspired by the upper-limb system, this work revisits motion decoupling in tendon-driven humanoid arms from a biomimetic mechanism design perspective. A tendon-driven architecture is proposed that achieves mechanical decoupling among multiple joints, enabling full motor–link separation while maintaining low mass and high compliance. From a biomimetic perspective, decoupled joint space actuation represents a functional abstraction of human muscle organization. Accordingly, each degree of freedom of the proposed tendon-driven arm is actuated by an antagonistic tendon pair. This decoupled multi-degree-of-freedom structural design enables high positioning accuracy while reducing link mass and improving overall system integration.

As shown in Table 1, prior tendon-driven humanoid arms inevitably trade motor–link separation for joint space decoupling, or require control-based compensation to suppress distal tendon interference. This work resolves this trade-off and achieves full motor–link separation and inherent joint space decoupling without control compensation while simultaneously achieving high positioning repeatability.

The main contributions of this work are summarized as follows:

1. A fully decoupled tendon-driven humanoid arm (FDTDH-Arm) is proposed which achieves inherent motion decoupling among multiple joints and enables complete motor–link separation.
2. The forward and inverse kinematic models of the proposed joint architecture and the entire tendon-driven manipulator are established, and the decoupling conditions for a single elbow joint and shoulder joint are analyzed.
3. A prototype of the proposed FDTDH-Arm is developed, and comprehensive experiments are conducted to validate its performance.

The rest of this article is organized as follows: Section 2 presents the mechanism design of the FDTDH-Arm. Section 3 establishes the forward and inverse kinematic models for the FDTDH-Arm. Section 4 reports the experimental validation, including joint-level validation and prototype performance. Section 5 discusses the experimental results and design. Finally, Section 6 concludes the article.

## 2. Mechanical Design

This section details the mechanical design of the proposed FDTDH-Arm, which is developed based on biomimetic principles inspired by the actuation mechanism of the human upper arm. The design emphasizes decoupled joint space actuation, antagonistic tendon-driven joints, and centralized actuation to achieve a lightweight structure, accurate motion, and high system integration.

### 2.1. Design Requirements and Biomimetic Principles

From a biomimetic perspective, the human upper limb provides an effective functional template in which joint motions are generated through antagonistic muscle–tendon coordination and regulated in a largely joint space- or task space-oriented manner. Although the musculoskeletal system exhibits substantial anatomical redundancy and multi-joint coupling, motor control can be abstracted as the functionally decoupled regulation of individual joint degrees of freedom.

Accordingly, the mechanical design is guided by the following requirements and biomimetic principles:

1. Joint space decoupling: each joint degree of freedom (DoF) is actuated through an independent antagonistic tendon pair to enable bidirectional torque generation and reduce inter-DoF coupling.
2. High compliance: the tendon-driven actuation and antagonistic configuration inherently provide mechanical compliance, enhancing safety and adaptability during physical interaction.
3. Lightweight and remote actuation: actuators are relocated away from the links to minimize mass and inertia, improving dynamic response and safety.
4. High system integration: all actuators are centralized within a compact drive unit, facilitating modular assembly, maintenance, and system integration.

### 2.2. Overall FDTDH-Arm Design

To translate the biomimetic design principles discussed in the previous subsection into a realizable mechanical system, this subsection introduces the overall design of the proposed robotic arm through a kinematic abstraction of the human upper limb. Rather than replicating anatomical structures directly, the design focuses on extracting the dominant functional degrees of freedom that are most relevant to upper-limb manipulation.

**Figure 1:**
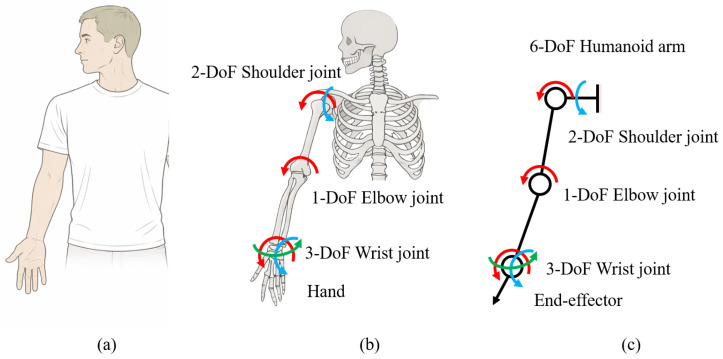
The implementation of the humanoid arm. The arrows indicate rotational degrees of freedom (DOF) at the arm. (**a**) Human upper limb. (**b**) Human upper-limb skeletal structure with kinematic abstraction. (**c**) Functional kinematic abstraction of joint DoF used in the robotic arm design.

As shown in Figure 1a, the human upper limb consists of complex skeletal structures and multiple anatomical joints. While these structures enable rich motion capabilities, they also exhibit substantial redundancy from an engineering perspective. To facilitate robotic implementation, the human upper limb is therefore interpreted at a functional level, where joint motions are characterized by a limited set of dominant DoFs governing task execution.

Based on this interpretation, Figure 1b illustrates the kinematic abstraction adopted in this work. Specifically, the shoulder is abstracted as a 2-DoF joint, representing its primary flexion–extension and abduction–adduction motions, which dominate arm positioning during manipulation tasks. The elbow is modeled as a 1-DoF hinge joint corresponding to flexion–extension, serving as the principal contributor to reach modulation. The wrist complex is abstracted as a 3-DoF joint, capturing wrist flexion–extension, radial–ulnar deviation, and axial rotation, which collectively determine the end effector position and orientation. Following this abstraction, the proposed robotic arm is designed with a total of six DoFs. This design establishes a direct correspondence between the abstracted human joint motions and the robotic joint degrees of freedom, enabling the robotic arm to reproduce the principal kinematic functionalities of the human upper limb while maintaining mechanical simplicity.

Notably, certain anatomical degrees of freedom, such as complex scapulothoracic motions, are intentionally omitted in this abstraction. This choice reflects a functional biomimetic strategy that prioritizes task-relevant motion generation and controllability over anatomical completeness. Based on this kinematic abstraction, the following subsection introduces the decoupled joint space actuation architecture employed for each DoF.

### 2.3. Decoupled Joint Space Actuation Design and Modeling

Building upon the kinematic abstraction introduced in the previous subsection, this subsection presents the decoupled joint space actuation architecture adopted for the proposed robotic arm. The design aims to realize independent and bidirectional torque generation for each joint DoF while eliminating inter-DoF coupling and preserving a lightweight structure.

#### 2.3.1. Actuation Principle and Decoupling Conditions

To realize decoupled joint space actuation in a tendon-driven multi-DoF arm, the actuation tendons associated with each joint are functionally classified into two categories; tendons that directly generate torque for the motion of a given joint are referred to as local actuation tendons (LATs), whereas tendons that pass through the joint to transmit actuation to distal joints are referred to as distal transmission tendons (DTTs).

From a kinematic perspective, the length variation of a tendon routed through a joint can be expressed as a function of the joint motion. For a joint with generalized coordinate *q*, the tendon length variation $\Delta l_{i}$ of the *i*-th tendon can be written in the general form

(1) $$\Delta l_{i}=f_{i}\left(q\right),$$

where $f_{i}\left(\cdot \right)$ denotes the tendon–joint mapping determined by the routing and winding geometry. For LATs, this mapping is intentionally designed such that the tendon length variation is directly related to the corresponding joint motion, enabling effective torque generation along the joint DoF.

In contrast, DTTs should transmit actuation to distal joints without being affected by the motion of the current joint. Accordingly, joint space decoupling requires that the tendon length variations of DTTs be insensitive to the joint motion. This requirement can be expressed by the following decoupling condition:

(2) $$\frac{\partial \Delta l_{\mathrm{DTTs}}}{\partial q}\approx 0,$$

which ensures that the motion of the joint does not introduce unintended actuation or disturbance to distal joints at the kinematic level.

The above actuation principle and decoupling condition constitute a joint-independent theoretical framework for the proposed architecture. By appropriately designing the tendon routing and winding geometry to satisfy these conditions, local joint actuation and distal transmission can be functionally separated within a unified tendon-driven system. In the following subsections, this framework is instantiated for individual joints, starting from the single-DoF elbow joint and extending to the multi-DoF wrist and shoulder joints.

#### 2.3.2. Elbow Joint Actuation

**Figure 2:**
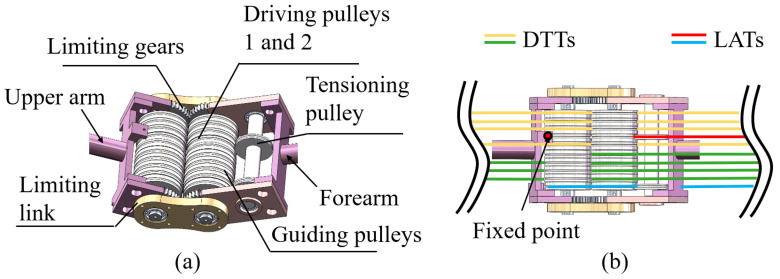
Elbow joint actuation configuration. (**a**) Assembly. (**b**) Tendon routing of the DTTs and LATs.

As a single-DoF joint, the elbow provides the simplest and most representative instantiation of the actuation principle and decoupling conditions introduced above. Based on these conditions, a joint configuration of the elbow actuation is schematically shown in Figure 2.

In the elbow joint, a gear with a module of 1 and 40 teeth is employed to enforce a pure rolling constraint between adjacent links. The guide pulleys used for decoupling are designed with diameters equal to the pitch circle diameter and are mounted on stepped shafts with embedded ball bearings. The driving pulley assembly consists of three pulleys, as shown in Figure 2a, including one tensioning pulley and two guide pulleys. The tensioning pulley has a diameter of 20 mm, while driving pulleys 1 and 2 both have diameters of 39.5 mm. These pulleys are used to route the LATs to fixed anchoring points, where the tendons are secured using aluminum sleeves. The top view in Figure 2b illustrates the spatial arrangement of the DTTs and LATs, as well as the locations of the tendon anchoring points.

**Figure 3:**
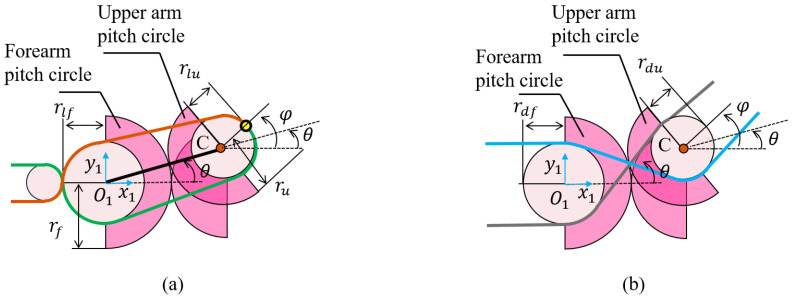
Equivalent configurations of the elbow joint actuation. (**a**) Configuration for deriving the transmission ratio of LATs. (**b**) Configuration for deriving the decoupling condition of DTTs.

To provide a geometric interpretation of the transmission relationship and the decoupling requirement, an equivalent configuration of the elbow joint is presented in Figure 3, which abstracts the routing geometry that governs tendon length variations.

As illustrated in Figure 3a, the two pitch circles roll in a manner that is purely relative to each other. The radii of the pitch circles are denoted as $r_{f}$ and $r_{u}$, respectively. Thus, the relationship between their arc lengths is given by

(3) $$r_{f}\theta =r_{u}\left(\varphi -\theta \right),$$

where φ is the rotation angle of the rod, and θ is the rotation angle of the link between the two pitch circles.

By factoring, the relationship between φ and θ is given by

(4) φ=γθ,

where $\gamma =\left(r_{f}+r_{u}\right)/r_{u}$.

The LATs form an antagonistic pair that generates bidirectional joint torque. On the motor side, the two tendons are wound on a common tendon spool with an identical radius $r_{t}$, resulting in symmetric antagonistic length variations. Therefore, the transmission ratio can be derived by analyzing only one tendon without loss of generality.

For a single side tendon, the variation in tendon length can be calculated by

(5) $$\Delta l_{t}=\theta_{t}r_{t},$$

where $\Delta l_{t}$ is the variation in tendon length, and $\theta_{t}$ is the rotation angle of the tendon spool.

Without loss of generality, $\Delta l_{t}$ is assumed to be positive. Considering guide pulleys of the forearm and upper arm with radii $r_{lf}$ and $r_{lu}$, respectively, the wrap angle of the forearm guide pulley increases by θ, while the wrap angle of the upper-arm guide pulley increases by φ−θ. Since the change in the joint tendon length is entirely induced by the variation in the tendon length on the motor side, the relationship can therefore be derived as

(6) $$\begin{array}{cc} \Delta l_{c} & =\theta r_{lf}+\left(\varphi -\theta \right)r_{lu}\\ \; & =\theta \left(r_{lf}-r_{lu}\right)+\theta \gamma r_{lu} \end{array}.$$

Substituting (5) into (6), we obtain the transmission ratio *i* as

(7) $$i=\frac{\theta }{\theta_{t}}=\frac{r_{t}}{r_{lf}+\left(\gamma -1\right)r_{lu}}.$$

Having determined the transmission ratio, we proceed to derive the decoupling conditions. To realize the decoupling function, the tendons driving the distal joint are crossed and wound around a set of guide pulleys, as shown in Figure 3b. Similarly, the variation of the wrap angle of each pulley is analyzed, from which the total tendon length variation is derived.

During motion, the relationship between the rotation angle θ and the tendon length variation of the forearm guide pulley can be expressed as

(8) $$\Delta l_{df}=r_{df}\theta ,$$

where $\Delta l_{df}$ is the tendon length variation corresponding to the change in the forearm pulley wrap angle, and $r_{df}$ is the radius of the forearm’s guide pulley.

Similarly, for the upper-arm guide pulley, we derive

(9) $$\begin{array}{cc} \Delta l_{du} & =-r_{du}\left(\varphi -\theta \right)\\ \; & =-r_{du}\left(\frac{r_{f}+r_{u}}{r_{u}}\theta -\theta \right)\\ \; & =-r_{du}\frac{r_{f}}{r_{u}}\theta , \end{array}$$

where $\Delta l_{du}$ is the tendon length variation corresponding to the change in the upper-arm pulley wrap angle, and $r_{du}$ is the radius of the upper-arm guide pulley.

The key to achieving decoupling is maintaining an invariant tendon length during joint rotation. Thus, the relationship between $\Delta l_{df}$ and $\Delta l_{du}$ is

(10) $$\Delta l_{df}+\Delta l_{du}=0.$$

According to (8)–(10), the decoupling condition is obtained as

(11) $$\frac{r_{df}}{r_{du}}=\frac{r_{f}}{r_{u}}.$$

Equation (11) indicates that, to achieve kinematic decoupling, the radius ratio of the forearm and upper-arm decoupling pulleys must match the ratio of their respective joint pitch circles. In the proposed FDTDH-Arm, $r_{f}=r_{u}=20$ mm, $r_{lf}=r_{lu}=19.75$ mm, and $r_{df}=r_{du}=20$ mm, thereby satisfying the decoupling condition.

#### 2.3.3. Wrist Joint Actuation

The wrist joint adopts an actuation architecture that is structurally analogous to the elbow joint while extending the decoupled joint space actuation principle to a multi-degree-of-freedom configuration. Specifically, the wrist is realized by serially cascading two elbow-like actuation modules arranged in orthogonal directions, thereby enabling decoupled motion generation along multiple rotational axes.

**Figure 4:**
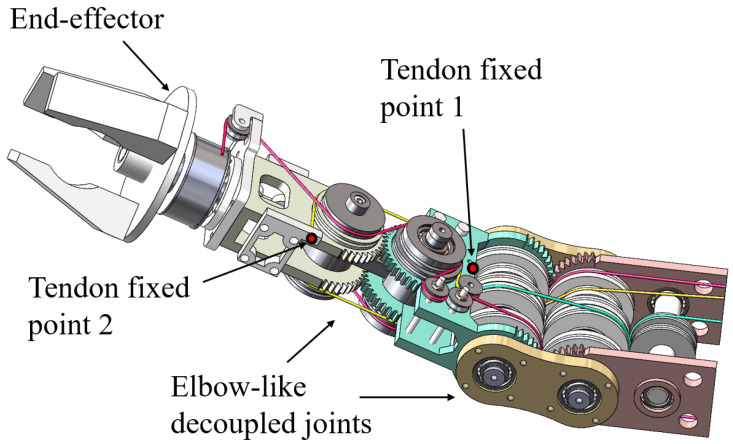
The configurations of the wrist joint.

As shown in Figure 4, from a joint space perspective, the wrist joint comprises two perpendicular single-DoF actuation modules, each following the same actuation principle and decoupling conditions established for the elbow joint. Each module is actuated by an independent antagonistic pair of LATs to generate bidirectional torque about its corresponding axis, while DTTs traverse the joint without introducing kinematic coupling. Leveraging this modular arrangement, the actuation of each wrist degree of freedom can be regulated independently in joint space.

In addition to the two DoFs, the wrist incorporates a rotational DoF along the tool axis to achieve full three-axis orientation capability. By constructing the wrist joint as a serial combination of orthogonally arranged decoupled actuation modules, the proposed design extends the single-joint decoupling framework to a compact multi-axis joint. This approach enables a scalable and modular wrist design while maintaining the lightweight, compliant, and decoupled properties of the overall tendon-driven arm.

#### 2.3.4. Shoulder Joint Actuation

The shoulder joint provides the primary positioning capability of the arm and is characterized by higher kinematic complexity than the elbow and wrist joints. Rather than replicating the full anatomical structure of the human shoulder, the proposed design adopts a functional abstraction in which the shoulder is implemented as a combination of two decoupled rotational degrees of freedom. Each degree of freedom follows the same joint space actuation principle and decoupling conditions established for the elbow joint, enabling independent torque generation while eliminating inter-joint coupling.

Despite the apparent similarity to the elbow and wrist joints, the shoulder joint introduces additional challenges due to its higher actuation density and functional requirements. First, the two shoulder degrees of freedom must be mutually decoupled to enable independent torque generation along each rotational axis. Second, the tendon transmission to distal joints must remain invariant to both shoulder motions, such that joint space decoupling is preserved throughout the kinematic chain. These compounded constraints significantly increase the complexity of tendon routing and joint configuration design compared with single-DoF joints.

**Figure 5:**
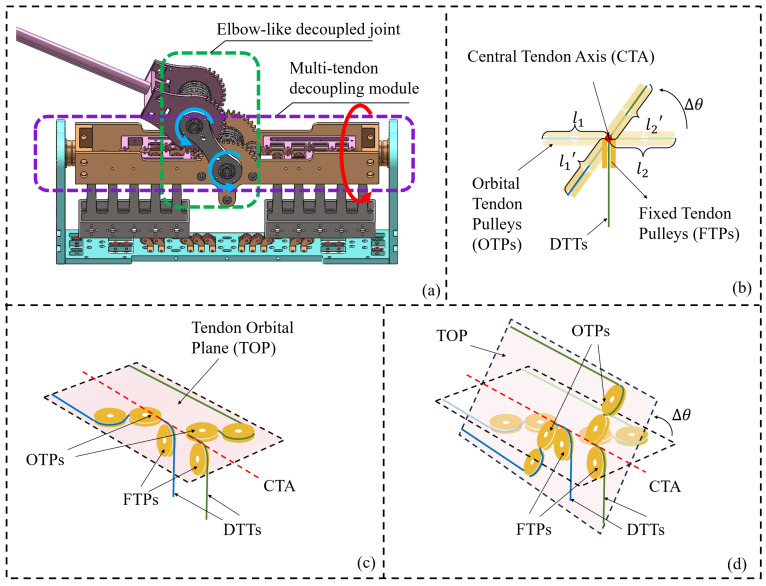
The configuration and decoupling method of the shoulder joint. The red and blue arrows denote the first and second degrees of freedom of the shoulder, respectively. (**a**) Configuration of the shoulder joint. (**b**) View along the normal plane of the Central Tendon Axis (CTA), illustrating constant tendon length during joint rotation. (**c**) MTDM before rotation. (**d**) MTDM after rotation.

To address these challenges, a multi-tendon decoupling module (MTDM) is designed to achieve decoupling of five antagonistic DTT pairs. This module is then serially combined with an elbow-like joint to form a 2-DoF decoupled shoulder joint, as shown in Figure 5a.

The decoupling principle of the proposed multi-tendon decoupling module is explained by examining the geometric relationship between tendon routing and joint motion. As illustrated in Figure 5c, the pulley set comprises two types of elements: fixed tendon pulleys (FTPs) and orbital tendon pulleys (OTPs). The FTPs are arranged to be tangent to the Central Tendon Axis (CTA), ensuring that the tendons remain aligned with the axis during joint motion, whereas the OTPs are distributed within a tendon orbital plane (TOP) defined with respect to the CTA.

As shown in Figure 5b, which presents a view along the CTA (normal to the TOP), the tendon endpoints trace circular trajectories around the CTA. The radius of each trajectory is equal to the perpendicular distance from the outer edge of the corresponding OTP to the CTA, thereby ensuring that the tendon lengths remain invariant with respect to the joint rotation.

#### 2.3.5. Centralized Actuation Unit

To support the decoupled joint space actuation architecture described above, all actuators are centralized within a compact actuation unit located at the base of the arm. By integrating multiple motors and transmission components into a single, densely packaged module, this compact design enables the remote actuation of individual joints via tendons while minimizing the overall system volume. Consequently, the mass and inertia of the moving links are significantly reduced, leading to improved dynamic performance and enhanced safety during interaction.

### 2.4. Overall System Assembly

**Figure 6:**
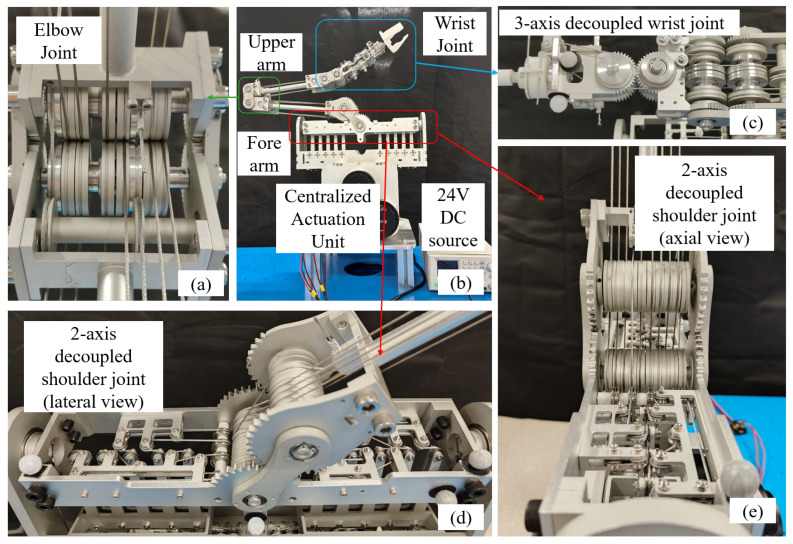
Prototype of the proposed fully decoupled tendon-driven humanoid arm. (**a**) Elbow joint actuation module. (**b**) Full system overview, including the centralized actuation unit, upper arm, forearm, elbow joint, and wrist joint. (**c**) Three-axis decoupled wrist joint. (**d**) Two-axis decoupled shoulder joint (lateral view). (**e**) Two-axis decoupled shoulder joint (axial view).

An overview of the proposed tendon-driven arm is shown in Figure 6. The system is composed of a centralized actuation unit, a shoulder joint, an elbow joint, and a wrist joint arranged in a serial kinematic chain. This overall assembly illustrates how the joint-level decoupled actuation modules described in the previous subsections are integrated into a compact and coherent robotic arm.

Owing to the centralized actuation and decoupled transmission design, the total mass of the moving arm structure is minimized to 1.90 kg, thereby achieving a lightweight configuration suitable for humanoid manipulation.

## 3. Kinematic Analysis

This section presents the kinematic analysis of the proposed tendon-driven arm to demonstrate how the mechanically enforced joint space decoupling facilitates motion modeling and control. Building upon the kinematic abstraction and decoupled actuation architecture described in Section 2, the analysis is conducted at both the joint level and the whole-arm level. Specifically, joint-level kinematics are first examined to highlight the motion characteristics of elbow-like joints, followed by forward and inverse kinematic modeling of the entire arm. Finally, the reachable workspace of the arm is analyzed to evaluate its motion capability in humanoid manipulation tasks.

### 3.1. Joint-Level Kinematic Modeling

Due to the composite joint structures of the proposed arm, directly applying the standard Denavit–Hartenberg (DH) convention would require introducing multiple intermediate coordinate frames that do not correspond to independent joint degrees of freedom. This would lead to unnecessarily long kinematic chains and obscure the functional decoupling inherent in the mechanical design. Therefore, the kinematic analysis is conducted from a joint-level perspective, where each mechanically decoupled joint is modeled as a single functional unit, and a tailored homogeneous transformation formulation is adopted to reduce the number of coordinate frames while preserving the kinematic equivalence.

**Figure 7:**
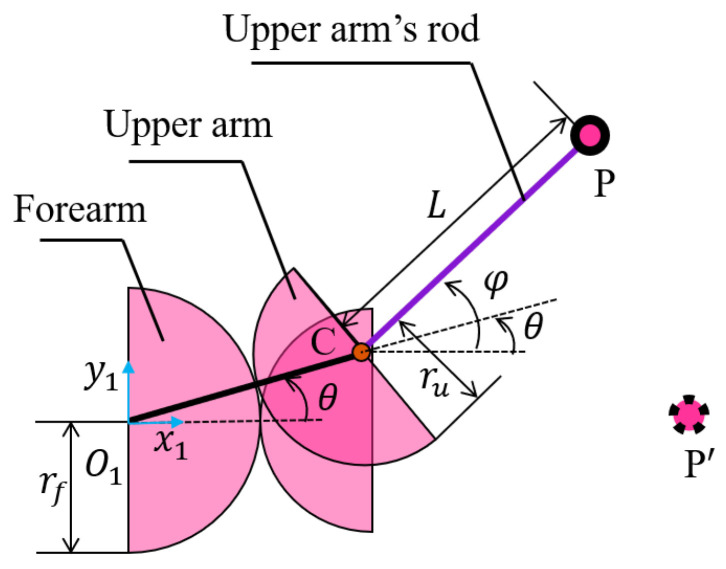
Kinematic abstraction of an elbow-like joint.

A single elbow-like joint is considered to analyze its kinematic model. Since the gears are used as pure rolling constraints, the joint can be abstracted as two pitch circles rolling relative to each other. As illustrated in Figure 7, a Cartesian coordinate system $O_{1}$ is defined at the center of the forearm pitch circle, with the $x_{1}$-axis aligned with the direction of the forearm. The center of the upper-arm pitch circle is located at

(12) $$C\left(\theta \right)=\left(r_{f}+r_{u}\right){\left(\cos\theta ,\sin\theta \right)}^{T},$$

where C(θ) denotes the center of the upper-arm pitch circle, and $r_{f}$ and $r_{u}$ represent the radii of the forearm and upper-arm pitch circles, respectively.

The vector from point C to the endpoint of the rod P is ${\left(L\cos\varphi ,L\sin\varphi \right)}^{T}$. Thus, the position of the point P(x(θ),y(θ)) in the coordinate system $O_{1}$ is

(13) $$\begin{array}{c} \left\{\begin{array}{c} x\left(\theta \right)=\left(r_{f}+r_{u}\right)\cos\theta +L\cos\left(\gamma \theta \right)\\ y\left(\theta \right)=\left(r_{f}+r_{u}\right)\sin\theta +L\sin\left(\gamma \theta \right). \end{array}\right. \end{array}$$

The motion of point P in the Cartesian coordinate system $O_{1}$ is the superposition of two circular components: the first is a circular motion with radius $\left(r_{f}+r_{u}\right)$, which determines the overall rotational trend of the trajectory; the second is an additional circular component of length *L*, whose angular displacement is $\theta \left(r_{f}+r_{u}\right)/r_{u}$.

Since θ is a time-varying variable, it can be expressed as a function of time. By differentiating the *x* and *y* components of P, the velocity Jacobian can be obtained as

(14) $$v=S\left(\theta \right)\dot{\theta }\;S\left(\theta \right)=\left(\begin{array}{c} -\left(r_{f}+r_{u}\right)\sin\theta -L\gamma \sin\left(\gamma \theta \right)\\ \left(r_{f}+r_{u}\right)\cos\theta +L\gamma \cos\left(\gamma \theta \right) \end{array}\right),$$

where v is the velocity of the point P, and S(θ) is the velocity Jacobian of the point P.

The drive tendons are coaxially routed, resulting in an antagonistic variation in tendon lengths. The transmission ratio was derived in Section 2.

### 3.2. Manipulator Kinematics Modeling

#### 3.2.1. Forward Kinematics

**Figure 8:**
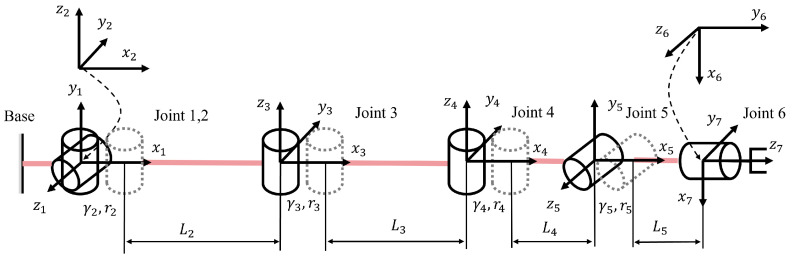
Schematic of the kinematic model of the FDTDH-Arm.

Following the kinematic analysis of a single joint, we further consider the case where multiple elbow-like joints are connected in series. The schematic of the kinematic model of the FDTDH-Arm is illustrated in Figure 8.

The homogeneous transformation matrix defines the mapping between two coordinate frames. In the proposed elbow-like joints, directly employing homogeneous transformations from the base frame to the guiding link frame provides an effective modeling framework. Taking the *i*-th joint as the base frame, the homogeneous transformation matrix of the link endpoint with respect to joint *i* is formulated as

(15) $$T_{i}=\left[\begin{array}{cccc} c\left(\gamma_{i}\theta_{i}\right) & -s\left(\gamma_{i}\theta_{i}\right) & 0 & r_{i}\gamma_{i}c\left(\theta_{i}\right)+L_{i}c\left({\gamma_{i}\theta }_{i}\right)\\ s\left(\gamma_{i}\theta_{i}\right) & c\left(\gamma_{i}\theta_{i}\right) & 0 & r_{i}\gamma_{i}s\left(\theta_{i}\right)+L_{i}s\left({\gamma_{i}\theta }_{i}\right)\\ 0 & 0 & 1 & 0\\ 0 & 0 & 0 & 1 \end{array}\right],$$

where $T_{i}$ represents the homogeneous transformation matrix of the *i*-th degree of freedom. Here, $\gamma_{i}$ denotes the pitch circle coefficient, $r_{i}$ is the upper-arm pitch circle radius, and $L_{i}$ is the rod length of the *i*-th joint. Additionally, we define c(θ)=cos(θ) and s(θ)=sin(θ) for notational brevity.

In practice, within a serial arrangement, the rotation axis of the preceding joint may form an angle $\alpha_{i}$ with that of the succeeding joint, or there may exist a joint offset $d_{i}$. Therefore, the resulting secondary transformation matrix is

(16) Tii−1=Ti(γ,ri,Li,θi)·Tx(di)·Rz(αi),

where $d_{i}$ and $\alpha_{i}$ represent geometric offsets between successive joint modules.

Then, the forward kinematics of the tendon-driven manipulator can be described as

(17) M(θ)=∏i=1jTii−1,

where M(θ) denotes the homogeneous transformation matrix from the base to the end effector, *j* denotes the total number of links, and $\theta ={\left[\theta_{1},\theta_{2},\ldots ,\theta_{j}\right]}^{T}$ denotes the joint angle vector.

Thus, the forward kinematic model of the entire manipulator has been derived. With γ=1 defined for a single revolute joint, the kinematic parameters of the proposed FDTDH-Arm are summarized in Table 2.

#### 3.2.2. Inverse Kinematics

Due to the presence of multiple intermediate links and the angular coupling introduced by the pure rolling constraints in the elbow-like joints, the forward kinematics of the proposed arm involve complex composite trigonometric relationships. Consequently, deriving a closed-form inverse kinematic solution is analytically intractable [28]. Therefore, a numerical inverse kinematics approach based on the damped least-squares (DLS) method is adopted.

The proposed arm features six actively controllable revolute joints, providing full six-degree-of-freedom controllability of the end effector pose in the six-dimensional task space (three translational and three rotational components). In human–robot collaboration scenarios, however, different components of the end effector pose often exhibit unequal task relevance. In particular, rotation about the tool axis—typically governed by the sixth joint—often plays a secondary role compared with the end effector position and approach direction (e.g., the orientation of the tool’s primary axis). To reflect this task-specific priority, the inverse kinematics is formulated as a weighted least-squares problem that emphasizes accuracy in position and key orientation components while softly regulating the less critical rotational degree of freedom.

Specifically, the joint velocity vector is computed as

(18) $$\dot{\theta }={\left(J^{T}WJ+\lambda^{2}I\right)}^{-1}J^{T}W\;\dot{x},$$

where $J\in R^{6\times 6}$ denotes the geometric Jacobian matrix of the manipulator, $\dot{x}\in R^{6}$ is the desired end effector twist, λ>0 is a damping factor that ensures numerical stability near kinematic singularities, and I is the 6×6 identity matrix. The diagonal weighting matrix $W=\mathrm{diag}(w_{1},\ldots ,w_{6})$ assigns higher weights to translational components and the primary orientation axes (e.g., pitch and yaw) while assigning a lower weight to the roll about the tool axis, thereby allowing the solver to prioritize task-relevant motion. In this study, the weighting matrix is set to W=diag(1,1,1,1,1,0.1), and the damping factor is set to $\lambda^{2}=0.001$ to ensure numerical stability. The Jacobian matrix J(θ) is derived from the forward kinematic model presented in the previous subsection and relates joint velocities to end effector twist via

(19) $$\dot{x}=J\left(\theta \right)\;\dot{\theta }.$$

## 4. Experimental Validation

This section experimentally evaluates the proposed decoupled tendon-driven humanoid arm (FDTDH-Arm). Five experiments were conducted to validate the core mechanical properties and system-level performance, including joint space decoupling, joint stiffness characteristics, payload capacity, positioning repeatability, and end effector motion speed (refer to Video S1). Together, these experiments demonstrate the effectiveness of the proposed mechanically enforced decoupling architecture and its suitability for human-centered manipulation tasks.

### 4.1. Joint-Level Validation

#### 4.1.1. Joint Space Decoupling

To verify the mechanically enforced joint space decoupling capability of the proposed FDTDH-Arm, a dedicated experimental platform was developed, representing the first functional prototype of the FDTDH-Arm. The objective of this experiment was to examine whether joint motions introduce unintended actuation or disturbance in mechanically decoupled joints, without relying on active control compensation.

**Figure 9:**
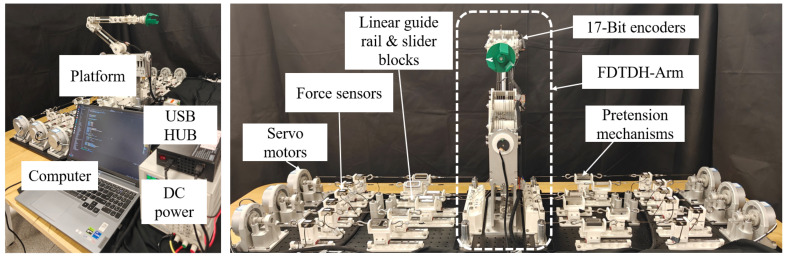
Experiment setup for decoupling validation.

The experimental platform mainly consisted of the FDTDH-Arm prototype and sensing modules, as shown in Figure 9. Six Unitree GM8010 motors were employed to generate tendon displacements, corresponding to the six joint degrees of freedom of the FDTDH-Arm. Each tendon was equipped with a tension–compression sensor (100 kg range) and a pretensioning mechanism to measure tendon tension and ensure proper tendon preload. To minimize frictional effects and approximate ideal transmission conditions, the sensors and pretensioning mechanisms were mounted on low-friction linear guide rails.

Due to the limited internal space within the arm links, 17-bit encoders were installed on Joints 1–4 only. The angular variation of Joint 4 was directly measured using the encoder, while the potential motion of Joint 6 was inferred from the corresponding tendon tension differences. This sensing configuration was sufficient for decoupling validation, since any unintended joint motion would necessarily appear as either a measurable angular deviation at Joint 4 or systematic tension imbalance in the distal transmission tendons.

Because of the structural homogeneity of the elbow-like joints adopted throughout the arm, the decoupling performance can be sufficiently evaluated by monitoring a representative joint. In this experiment, Joint 4 was selected as the observation joint. Two representative motion trajectories were executed for decoupling validation. In Trajectory 1, the motors of Joint 4 and Joint 6 were commanded to hold a fixed position. In Trajectory 2, the motors of Joint 4, Joint 5, and Joint 6 were commanded to hold a fixed position.

**Figure 10:**
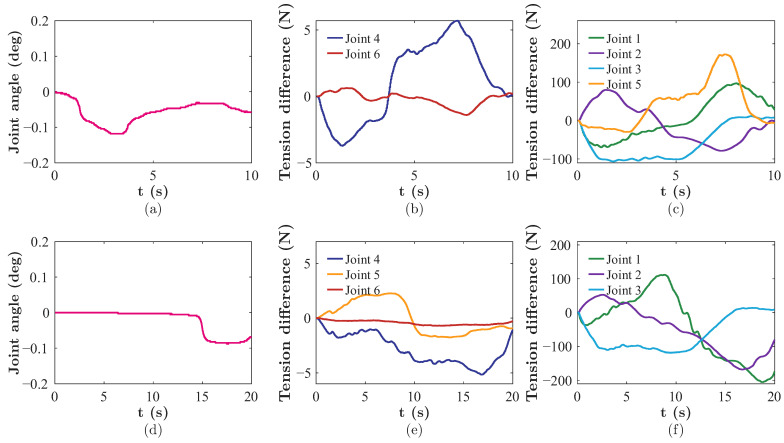
Experimental results for the two trajectories. (**a**) Induced motion of Joint 4 over time in Trajectory 1. (**b**) Tendon tension difference of Joint 4 and Joint 6 over time in Trajectory 1. (**c**) Tendon tension difference of active joints over time in Trajectory 1. (**d**) Induced motion of Joint 4 over time in Trajectory 2. (**e**) Tendon tension difference of Joint 4, Joint 5, and Joint 6 over time in Trajectory 2. (**f**) Tendon tension difference of active joints over time in Trajectory 2.

As shown in Figure 10a,d, the angular variation of Joint 4 remained strictly bounded, with an overall mean of $0.0373^{\circ }\pm 0.0372^{\circ }$ (mean ± std) across all sampled data points. Conversely, Figure 10b,e indicate that the corresponding tendon tension difference for Joint 4 exhibited noticeable fluctuations (ranging from approximately −4 N to 6 N). This phenomenon can be attributed to the inherent friction within the pulleys and joints, which counteracts minor disturbance torques.

For Joint 6 across both trajectories, the coupling effect was even weaker, with the measured tendon tension difference varying within a narrower range (approximately −2 N to 3 N). Similarly, for Joint 5 in Trajectory 2, the coupling effect was also weaker than that of Joint 4 (approximately −3 N to 3 N).

When compared to the actively driven joints, which require large tension differences to generate motion (see Figure 10c,f), the tension differences in the distal joints were bounded within a minimal range (below ±6 N). This value is orders of magnitude lower than that of the driven joints. This magnitude comparison establishes a physical baseline, demonstrating that the minor tension fluctuations in the distal joints are insufficient to overcome the static friction threshold of the transmission system. Consequently, the induced motion of the distal joints is effectively negligible.

Overall, the results indicate that the proposed arm realizes effective joint space decoupling through its mechanically inspired architecture, enabling independent joint regulation in a manner consistent with functional abstractions of human motor organization.

#### 4.1.2. Joint Stiffness Characteristics

Mechanical compliance is a key property for the FDTDH-Arm, intended for human-centered interaction, as it directly affects safety, adaptability, and contact behavior. A commonly adopted metric to quantify mechanical compliance is joint stiffness, which characterizes the relationship between an externally applied torque and the resulting angular displacement. In the proposed tendon-driven arm, joint stiffness is primarily governed by the antagonistic tendon transmission and rolling-based joint mechanism rather than by high-ratio gear trains. Therefore, evaluating the joint stiffness provides direct insight into the inherent compliance of the mechanical design.

**Figure 11:**
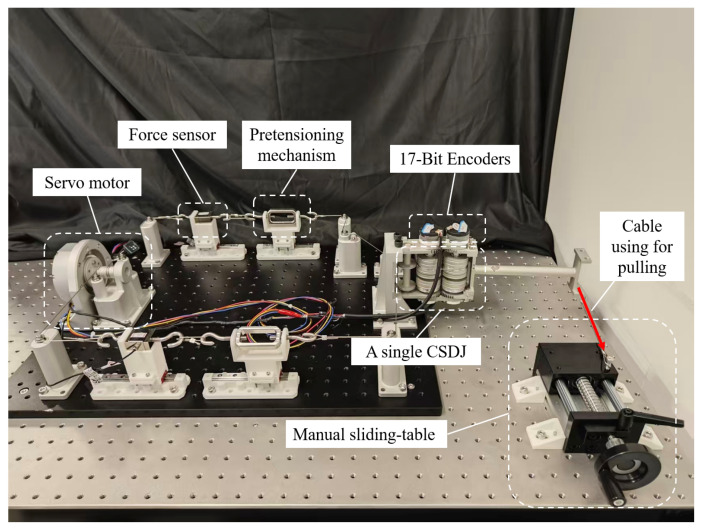
Experimental setup for testing elbow-like joint stiffness.

To characterize the stiffness behavior of the proposed elbow-like joint, a dedicated joint-level experimental platform was developed, as shown in Figure 11. The platform consisted of a single elbow-like joint, a driving unit, and sensing components. Two tension–compression sensors (100 kg range) were mounted on the driving tendons to measure tendon tension differences, and two angle encoders were installed to record the joint angle. In addition, a manual sliding-table module equipped with a handwheel was used to apply external disturbance torques to the joint in a controlled manner. By adjusting the handwheel, small angular displacements were induced while tendon tensions and joint angles were continuously recorded.

Based on the measured tendon tension difference $\Delta F_{\mathrm{read}}$, the joint torque can be calculated as

(20) $$\tau_{\mathrm{read}}=\Delta F_{\mathrm{read}}\left(r_{\mathrm{pulley}1}+\left(\gamma -1\right)r_{\mathrm{pulley}2}\right),$$

where $r_{\mathrm{pulley}1}$ and $r_{\mathrm{pulley}2}$ denote the radii of the forearm and upper-arm guide pulleys, respectively. Using the encoder-measured joint angle θ and the corresponding torque $\tau_{\mathrm{read}}$, a least-squares linear fitting is applied to the torque–angle relationship. The slope of the fitted line is taken as the effective joint stiffness at the tested configuration.

To evaluate the compliance characteristics of the FDTDH-Arm, static stiffness measurements were conducted at three distinct joint angles ($0^{\circ }$, $45^{\circ }$, and $90^{\circ }$) under varying antagonistic tendon tensions ranging from 50 N to 150 N.

**Figure 12:**
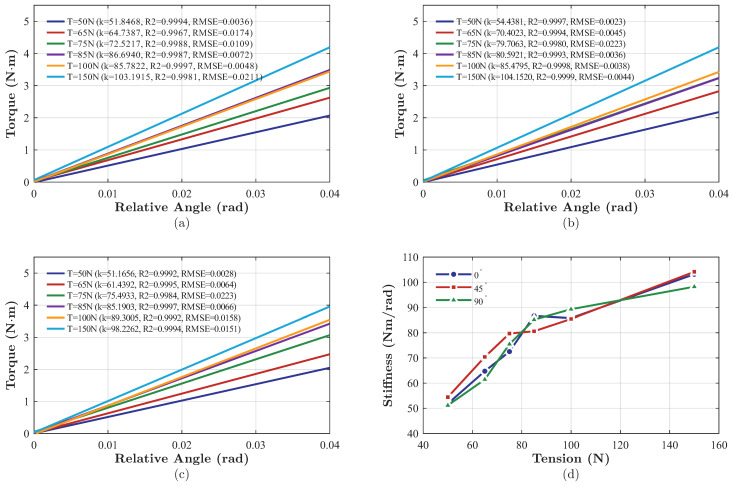
Experimental results for joint stiffness. (**a**) Angle–torque graph of the elbow-like joint at 0°. (**b**) Angle–torque graph of the elbow-like joint at 45°. (**c**) Angle–torque graph of the elbow-like joint at 90°. (**d**) Stiffness–tendon tension graph for 0°, 45°, and 90°.

The torque–angle relationships exhibited highly linear behavior across all tested joint angles. Given that the experimental results exhibited a predominant linear region, the stiffness characterization focuses exclusively on this linear segment. To provide a clear visualization of the trends under varying pretensions, only the linear fitted curves are plotted. Figure 12a–c present the stiffness measurement results under varying tendon pretensions at joint angles of $0^{\circ }$, $45^{\circ }$, and $90^{\circ }$, respectively. It can be observed that the joint stiffness exhibited a consistent trend regardless of the joint angle. The identified stiffness values are detailed in Table 3. The results indicate that joint stiffness is positively correlated with tendon tension. As shown in Figure 12d, the average stiffness increased from 52.48 N·m·rad^−1^ to 101.86 N·m·rad^−1^ as the tension rose from 50 N to 150 N. This stiffness range is of the same order of magnitude as the physiological stiffness of the human upper limb in task-constrained conditions (typically 15–200 N·m·rad^−1^) [29], indicating that the developed FDTDH-Arm possesses a certain degree of compliance beneficial for safe physical interaction.

### 4.2. Prototype Performance

To verify the performance of the proposed FDTDH-Arm, a prototype was developed to investigate its load capacity, repeatability, and motion speed. The arm body and the driving unit were made of aluminum alloy 6061. The prototype utilized deep-groove ball bearings to ensure low friction for each joint and pulley. All mechanical components were manufactured with an IT8 tolerance grade, and standard H7/g6 fits were employed for the deep-groove ball bearing assembly. Steel wire ropes (6×7 construction) with a diameter of 1.2 mm were used as tendons. Prior to experiments, each tendon was pretensioned to a target force of 80 N ± 20 N. Pretension was applied by pulling the tendon using a force gauge until the target range was reached, and was fixed by aluminum crimp sleeves.

**Figure 13:**
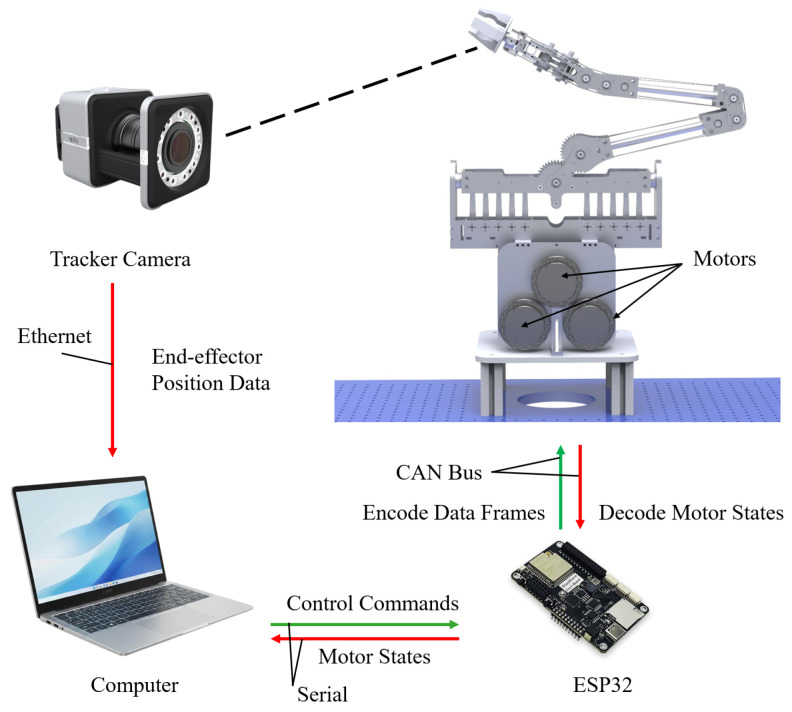
Overall system architecture and mechatronics circuit of the tendon-driven humanoid arm.

The prototype used HT8115-J6 motors as actuators, which feature integrated FOC (Field-Oriented Control) driver circuits. As shown in Figure 13, a computer served as the high-level controller for trajectory planning. An ESP32 control board acted as the real-time communication bridge, which receives commands from the computer via serial and transmits them to the motors via the CAN bus.

#### 4.2.1. End Effector Payload Capacity

End effector payload capacity reflects the mechanical strength and transmission robustness of the arm under external loading. A 2 kg payload was chosen as it corresponds to the upper range of typical objects encountered in daily human manipulation tasks, making the experiment representative of practical humanoid applications.

**Figure 14:**
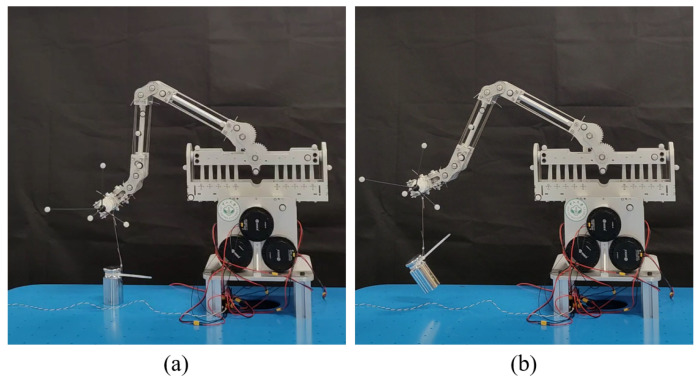
Experiment setup for the end effector payload capacity. The lifting height of the arm end effector is recorded using the VICON motion-capture system (measurement accuracy ±0.1 mm). (**a**) A 2 kg weight resting on the tabletop. (**b**) A 2 kg weight being lifted.

As shown in Figure 14, a 2 kg weight was selected as the test payload and placed on a tabletop. The end effector was commanded to execute a predefined lifting motion, and the vertical displacement of the end effector was recorded using a VICON motion-capture system with a measurement accuracy of ±0.1 mm. The same trajectory was repeated under unloaded and loaded conditions to assess the influence of payload on the lifting performance.

Under the 2 kg payload condition, the FDTDH-Arm successfully lifted the object from the tabletop and reached a maximum vertical displacement of 67.4 mm. The lifting motion remained stable and repeatable throughout the task. No abrupt velocity fluctuations, tendon slack-induced oscillations, or loss of stability were observed during the lifting process, indicating that the FDTDH-Arm maintains robust transmission behavior even under near-limit loading.

#### 4.2.2. Repeatability

**Figure 15:**
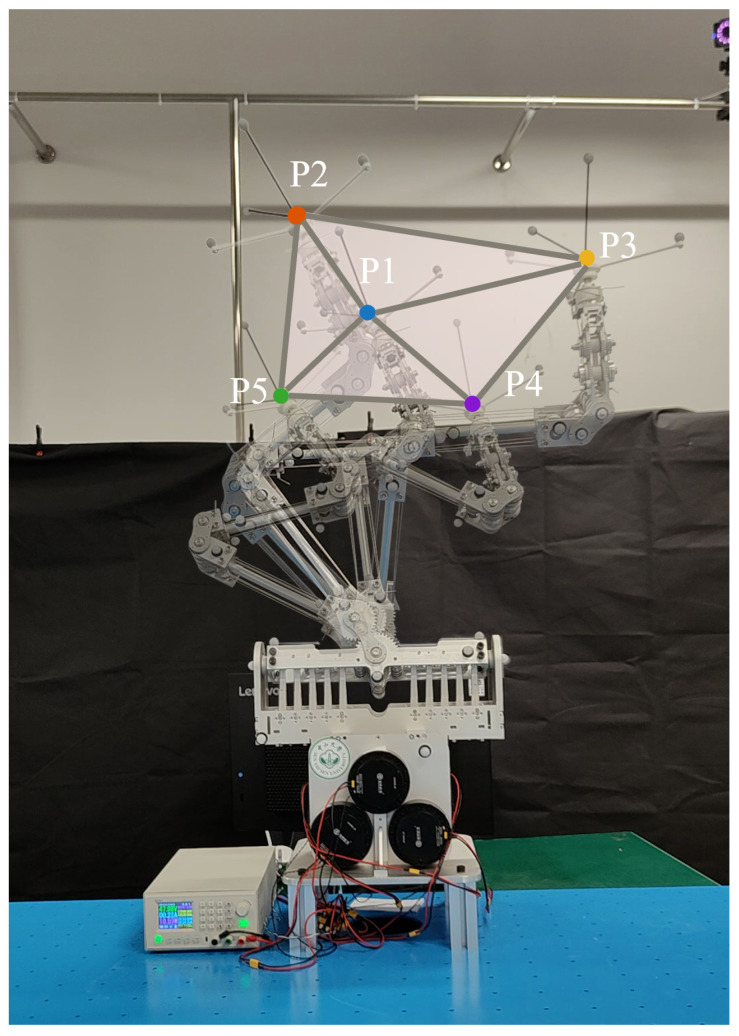
Experimental setup for repeatability test. Five predefined points (P1, P2, P3, P4, P5) were chosen to assess the repeatability of the proposed FDTDH-Arm, with the end effector executing 50 consecutive movements at each point. The position of each point is recorded using the VICON motion-capture system (measurement accuracy ±0.1 mm).

Repeatability was evaluated to assess whether the FDTDH-Arm can provide consistent end effector positioning, which is important in human–robot interaction. To evaluate the positioning repeatability of the proposed humanoid arm, a repeatability test was conducted following the statistical definition of position repeatability described in ISO 9283 [30], as shown in Figure 15. Position repeatability characterizes the dispersion of the end effector positions when the robot repeatedly executes the same motion toward an identical target under identical conditions, and reflects the intrinsic consistency of the mechanical structure, transmission, and control system.

The target positions were selected to span different regions of the reachable workspace, involving varying arm extensions and joint configurations, in order to assess repeatability under diverse kinematic conditions. For each target position, the same motion command was executed repeatedly for *n* trials under identical initial conditions. The three-dimensional Cartesian position of the end effector was recorded for each trial, yielding a set of measured positions $p_{i}=\left(x_{i},y_{i},z_{i}\right)$, where i=1,…,n.

Following ISO 9283, the barycentre (statistical center) of the measured positions was first computed as

(21) $$\bar{p}=\left(\bar{x},\bar{y},\bar{z}\right)=\frac{1}{n}\sum_{i=1}^{n}p_{i}.$$

The Euclidean distance between each measured position and the barycentre was then calculated as

(22) $$l_{i}=\sqrt{{\left(x_{i}-\bar{x}\right)}^{2}+{\left(y_{i}-\bar{y}\right)}^{2}+{\left(z_{i}-\bar{z}\right)}^{2}}.$$

The position repeatability RP is defined as

(23) $$RP=\bar{l}+3S_{l},$$

where $\bar{l}$ denotes the mean value of the distances $l_{i}$, and $S_{l}$ is their standard deviation. This metric corresponds to the radius of a sphere centered at the barycentre that statistically encloses approximately 99.7% of the measured positions, assuming a normal distribution.

Due to spatial limitations at the joints, joint-side encoders could not be installed. Therefore, the repeatability test was conducted using the motors’ internal closed-loop control. The actuators employed PD-based impedance control with feedforward torque. The parameters in this work were set to $K_{p}=300$, $K_{d}=1$. For the HT8115 motor, this residual internal friction was estimated to be $\tau_{res}\approx 0.1\;N\cdot m$. Based on these parameters, the steady-state error of the actuator was calculated as $\theta_{err}=\frac{\tau_{res}}{K_{p}}=\frac{0.1\;N\cdot m}{300\;N\cdot m/\mathrm{rad}}\approx 0.00033$ rad, which is sufficiently small to be considered negligible. Furthermore, a Trapezoidal Velocity Profile was employed for joint space trajectory planning. This strategy ensured continuous velocity and bounded acceleration, thereby preventing abrupt tension changes and the cable slackness inherent to the tendon-driven humanoid arm.

The repeatability results for the tested target positions are summarized in Table 4. The obtained mean positioning error $\bar{l}$ and repeatability RP values are 0.40 mm and 1.12 mm, respectively, demonstrating that the proposed humanoid robotic arm exhibited consistent and stable positioning performance across repeated trials. Despite the use of tendon-driven actuation and lightweight structural components, the system maintained a low dispersion of end effector positions, indicating good mechanical integrity and control repeatability. These results validate the suitability of the proposed design for tasks requiring reliable and repeatable positioning in human-centered and unstructured environments.

#### 4.2.3. Motion Speed

Motion speed characterizes the dynamic capability of the FDTDH-Arm and reflects its ability to execute rapid, coordinated whole-arm movements. For humanoid and human-interactive applications, such dynamic performance is particularly relevant, as many everyday actions involve fast arm motions rather than quasi-static positioning.

**Figure 16:**
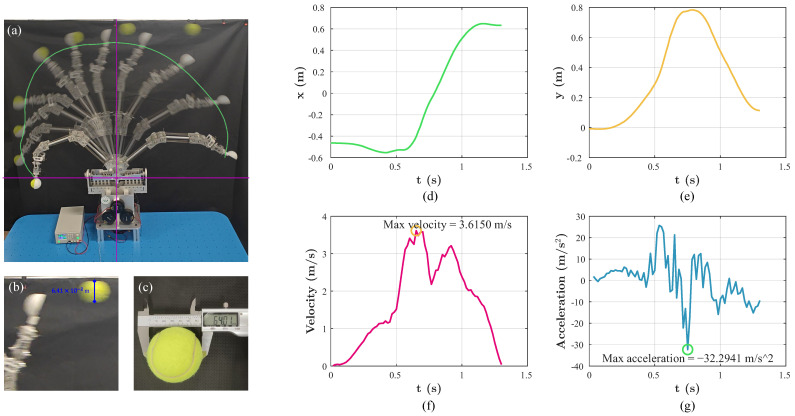
Experiment and results of the motion speed test. (**a**) A snapshot of the ball-throwing experiment, with the Cartesian coordinate frame and the end effector’s trajectory. (**b**) Calibration reference determined by the ball diameter at the release instant. (**c**) Actual diameter of the tennis ball. (**d**) End effector position along the *x*-axis as a function of time. (**e**) End effector position along the *y*-axis as a function of time. (**f**) End effector velocity profile during the throwing motion. (**g**) End effector acceleration profile during the throwing motion.

To evaluate the achievable end effector speed under dynamic conditions, a ball-throwing experiment was designed, as illustrated in Figure 16a–c. In this experiment, a tennis ball was released by the end effector during a fast swinging motion. The motion was recorded using a 1080p camera at 60 FPS and analyzed with the video analysis software Tracker 6.3. At the release instant, the translational velocity of the ball was assumed to be equal to the end effector velocity, which provides an effective approximation commonly adopted for estimating peak end effector speed in dynamic manipulation tasks.

The extracted velocity and acceleration profiles of the end effector are shown in Figure 16d,e. The FDTDH-Arm achieved a maximum end effector velocity of 3.62 m/s and a peak acceleration magnitude of 32.29 m/s^2^ during the throwing motion. These results indicate that, despite its compliant tendon-driven structure, the proposed FDTDH-Arm is capable of generating fast and explosive arm motions.

**Figure 17:**
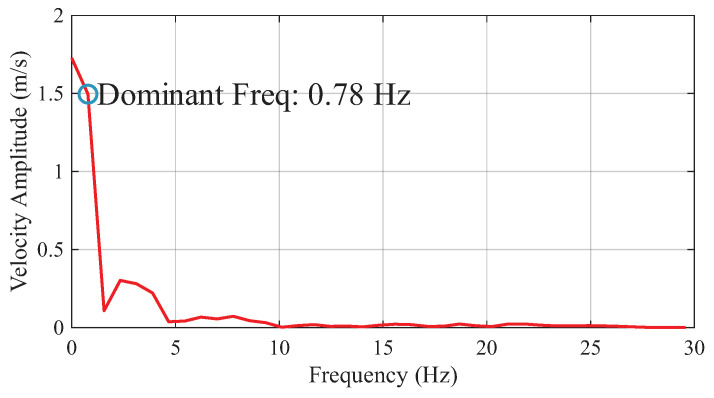
FFT analysis of the end effector velocity.

To further identify any dominant frequencies affecting the arm’s motion, a Fast Fourier Transform (FFT) analysis was performed on the end effector velocity data from the ball-throwing experiment. As shown in Figure 17, the spectrum reveals a clear dominant frequency at 0.78 Hz, which corresponds to the fundamental frequency of the throwing task itself. A minor secondary peak can be observed at 2.34 Hz, representing the third harmonic of the nonsinusoidal motion profile. There are no significant peaks in the higher frequency range (>5 Hz), which confirms that the FDTDH-Arm operates smoothly without structural resonance or parasitic vibrations, even during high-speed tasks.

Such dynamic performance demonstrates the effectiveness of the decoupled joint space actuation architecture in supporting high-speed motion without sacrificing mechanical compliance, highlighting the suitability of the proposed design for dynamic humanoid manipulation tasks.

## 5. Discussion

This study investigated the design of decoupled tendon-driven humanoid arms from the perspective of mechanically grounded joint space regulation. Rather than pursuing performance improvements through increasingly complex control strategies, the proposed design emphasizes enforcing joint decoupling at the mechanical level, thereby shaping the fundamental system behavior prior to control intervention.

A key implication of the experimental results is that robust joint-level decoupling can be achieved without control-based compensation. The decoupling validation experiments demonstrated that the proposed joint architectures effectively suppress the propagation of proximal and distal transmission effects along the kinematic chain. Such mechanical invariance simplifies joint control and enhances system robustness under dynamic conditions. By adopting these joint architectures, the FDTDH-Arm can be fully decoupled, which includes motor–link separation, and kinematics decoupled, which is particularly important for achieving lightweight design and reliable controllability in humanoid arms. In addition, the FDTDH-Arm adopting this decoupled architecture exhibited improved positioning repeatability.

The proposed design also involves several trade-offs. Enforcing joint decoupling through rolling constraints, pulley geometry, and a tendon transmission routine, especially in the shoulder joint, increases mechanical complexity and imposes higher demands for manufacturing precision. Moreover, the current prototype adopts a fixed mechanical stiffness determined by joint geometry and tendon pretension, without active stiffness modulation capability. Certain anatomical degrees of freedom, such as scapulothoracic motion, were intentionally omitted to prioritize mechanical simplicity and control tractability.

## 6. Conclusions

This paper presented a fully decoupled tendon-driven humanoid arm that realizes joint space independence through tendon transmission design. Inspired by functional abstractions of the human upper limb, each degree of freedom is actuated by an independent antagonistic tendon pair, while distal transmission effects are isolated along the kinematic chain. This architecture enables a lightweight, decoupled, and compliant system without relying on control-based compensation. Kinematic modeling and analysis were developed to capture the unique characteristics of the elbow-like rolling joints and their serial integration. Extensive experimental validation demonstrated effective joint space decoupling, human-comparable joint stiffness, stable payload handling, consistent positioning repeatability, and high-speed dynamic motion capability. Collectively, these results indicate that the proposed arm achieves a balanced combination of lightweight design, decoupling, compliance, and dynamic performance, providing a promising mechanical foundation for humanoid manipulation in human-centered and unstructured environments. Future work will focus on integrating proprioceptive sensing and adaptive control strategies to further exploit the mechanical decoupling and compliance of the proposed architecture.

## Figures and Tables

**Table 1:** Comparison with representative tendon-driven humanoid arms.

System	Motor–Link Separation	Joint Space Decoupling	Decoupling Method	Distal Tendon Interference	Control Compensation
Kim et al. [18]	Partial	Partial	Mechanical + control	Residual	Yes
Wang et al. [24]	Full (Bowden)	Yes (Bowden)	Bowden	Significant (Bowden friction)	Yes
Suzuki et al. [25]	Full	No	Control	Significant	Yes
Tanaka et al. [26]	Full	No	Control	Significant	Yes
Luo et al. [27]	Full	Partial	Mechanical + control	Residual	Yes (Wrist joint)
This article	Full	Full	Mechanical	Negligible	No

**Table 2:** Kinematic parameters of the proposed FDTDH-Arms.

Link	$\theta_{i}$ (deg)	$\gamma_{i}$	$d_{i}$ (mm)	$r_{i}$ (mm)	$L_{i}$ (mm)	$\alpha_{i}$ (deg)
1	$\theta_{1}$	1	0	0	0	0
2	$\theta_{2}$	2	0	35	300	−90
3	$\theta_{3}$	2	0	20	240	0
4	$\theta_{4}$	2	0	20	45	0
5	$\theta_{5}$	2	0	20	108	90
6	−90	1	0	0	0	0
7	$\theta_{6}$	1	0	0	0	0

**Table 3:** Results for joint stiffness (Nm/rad) under different tendon tensions and joint angles.

Joint Angle	Tendon Tension (N)					
	50	65	75	85	100	150
$0^{\circ }$	51.85	64.74	72.52	86.69	85.78	103.19
$45^{\circ }$	54.44	70.40	79.71	80.59	85.48	104.15
$90^{\circ }$	51.17	61.44	75.49	85.19	89.30	98.23
Average	52.48	65.53	75.91	84.16	86.85	101.86

**Table 4:** Position repeatability results evaluated according to ISO 9283.

Target Point	$\bar{l}$ (mm)	$S_{l}$ (mm)	RP (mm)
Point 1	0.60	0.31	1.53
Point 2	0.35	0.22	1.01
Point 3	0.37	0.24	1.08
Point 4	0.33	0.18	0.88
Point 5	0.36	0.25	1.11
Total	0.40	0.24	1.12

## Data Availability

Data is contained within the article. The original contributions presented in the study are included in the article. Further inquiries can be directed to the corresponding author.

## Supplementary Materials

The following supporting information can be downloaded at https://www.mdpi.com/article/10.3390/biomimetics11020141/s1, Video S1: Experiment Validation.

## Nomenclature

The following nomenclature is used in this manuscript:

$r_{f}$	Pitch circle radius of the forearm
$r_{u}$	Pitch circle radius of the upper arm
$r_{lf}$	Radius of forearm guide pulley for actuation tendons
$r_{lu}$	Radius of upper-arm guide pulley for actuation tendons
$r_{df}$	Radius of forearm decoupling pulley for transmission tendons
$r_{du}$	Radius of upper-arm decoupling pulley for transmission tendons
$r_{t}$	Radius of tendon spool at the motor side
*L*	Length of connecting rod in elbow-like joint
$L_{i}$	Rod length of t *i*-th joint
$d_{i}$	Offset between successive joint modules
$r_{i}$	Pitch circle radius of the upper arm at the *i*-th joint
$\alpha_{i}$	Angle between adjacent joint axes
$\theta_{i}$	Rotation angle of the *i*-th joint
γ	Pitch circle coefficient $\left(r_{f}+r_{u}\right)/r_{u}$
$\gamma_{i}$	Pitch circle coefficient of the *i*-th joint
θ	Joint rotation angle
ϕ	Rotation angle of the rolling rod
$\dot{\theta }$	Joint angular velocity
Δl	Tendon length variation
$\Delta l_{t}$	Tendon length variation at motor side
$\Delta l_{c}$	Tendon length variation at joint side
$\Delta l_{df}$	Tendon length variation at the forearm guiding pulley
$\Delta l_{du}$	Tendon length variation at the upper-arm guiding pulley
*k*	Equivalent joint stiffness
*i*	Transmission ratio
C(θ)	Center position of rolling pitch circle
P(x,y)	Cartesian position of joint endpoint
J	Geometric Jacobian matrix
W	Weighting matrix in inverse kinematics
I	Identity matrix
x	End effector pose vector in task space
$\dot{x}$	End effector twist in task space
$T_{i}$	Homogeneous transformation matrix of the *i*-th elbow-like joint
Tii−1	Homogeneous transformation matrix from i−1-th joint to i−1-th joint
M	Homogeneous transformation matrix from base to end effector
λ	Damping factor in inverse kinematics
$p_{i}$	End effector position in trial *i*
$\bar{p}$	Barycentre of measured positions
$l_{i}$	Distance between $p_{i}$ and $\bar{p}$
$\bar{l}$	Mean positioning error
$S_{l}$	Standard deviation
RP	Position repeatability (ISO 9283)
